# Graphene oxide-based rechargeable respiratory masks

**DOI:** 10.1093/oxfmat/itab003

**Published:** 2021-03-02

**Authors:** Stelbin Peter Figerez, Sudeshna Patra, G Rajalakshmi, Tharangattu N Narayanan

**Affiliations:** Tata Institute of Fundamental Research - Hyderabad, Sy. No. 36/P Serilingampally Mandal, Gopanapally Village, Hyderabad - 500046, India; Tata Institute of Fundamental Research - Hyderabad, Sy. No. 36/P Serilingampally Mandal, Gopanapally Village, Hyderabad - 500046, India; Tata Institute of Fundamental Research - Hyderabad, Sy. No. 36/P Serilingampally Mandal, Gopanapally Village, Hyderabad - 500046, India; Tata Institute of Fundamental Research - Hyderabad, Sy. No. 36/P Serilingampally Mandal, Gopanapally Village, Hyderabad - 500046, India

**Keywords:** triboelectricity, tribo-electric nanogenerator, graphene oxide, N95, respiratory mask, rechargeable mask

## Abstract

Respiratory masks having similar standards of ‘N95’, defined by the US National Institute for Occupational Safety and Health, will be highly sought after, post the current COVID-19 pandemic. Here, such a low-cost (∼$1/mask) mask design having electrostatic rechargeability and filtration efficiency of >95% with a quality factor of ∼20 kPa^−1^ is demonstrated. This filtration efficacy is for particles of size 300 nm. The tri-layer mask, named PPDFGO tri, contains nylon, modified polypropylene (PPY), and cotton nonwoven fabrics as three layers. The melt-spun PPY, available in a conventional N95 mask, modified with graphene oxide and polyvinylidene fluoride mixture containing paste using a simple solution casting method acts as active filtration layer. The efficacy of this tri-layer system toward triboelectric rechargeability using small mechanical agitations is demonstrated here. These triboelectric nanogenerator (TENG)-assisted membranes have high electrostatic charge retention capacity (∼1 nC/cm^2^ after 5 days in ambient condition) and high rechargeability even in very humid conditions (>80% RH). A simple but robust permeability measurement set up is also constructed to test these TENG-based membranes, where a flow rate of 30–35 L/min is maintained during the testing. Such a simple modification to the existing mask designs enabling their rechargeability *via* external mechanical disturbances, with enhanced usability for single use as well as for reuse with decontantamination, will be highly beneficial in the realm of indispensable personal protective equipment.

## INTRODUCTION

The severe acute respiratory syndrome coronavirus 2 (SARS-CoV-2) outbreak has made a significant impact on global health and economic conditions and its impacts on humanity will last for several years [1]. Social distancing, regular-interval sanitization and use of personal protective equipment (PPE) are the precautionary measures for human protection recommended by the World Health Organization. Facial respiratory mask is an integral part of any PPE [2]. In this scenario, various respiratory masks were proposed and designed by different research groups [3–8], apart from the well-established, commercially available masks such as ‘N95’ [3, 8]. Different easy to make *do it yourself* (DIY) masks are also proposed to protect respiratory systems from the direct virus attack, and such DIY masks of nonwoven fabrics of several layers are found to be efficient in stopping the particulate matter of size down to a few nanometers [4–6, 9]. The mechanism behind such masks is mainly based on size exclusion and inertial impaction [10]. The efficient working of such masks critically depends on the layering which would create a large pressure difference in space between the human nose and the external environment leading to breathing issues. Hence, an efficient filter against virus (size ∼0.120 μm, where the viral aerosols will be >∼0.120 μm) [11] needs to be attained with minimum layers. It is recommended to use a less layered (3–4 layered with a pressure difference of 5–8 mbar) mask having better filter efficacy toward aerosols (<5 µm) and droplets (>5 µm) for prolonged usage as a PPE. Further, particles of smaller size (<1 µm, where a typical size of virus such as SARS-CoV-2 is found to be in the range of 0.05–1 µm) [12] can slowly diffuse through the nonwoven fabrics-based masks, even those with several layers. Masks are not only studied in terms of their filtration efficiency (E = 1 − $\frac{\eta \left(\mathrm{aftermembrane}\right)}{\eta \left(\mathrm{beforemembrane}\right)}$ × 100, where η is the particle number density of a particular size), but also by quality factor, Q defined as −$\frac{\log\alpha }{\Delta P}w\mathrm{here}\;\alpha \;is\;\mathrm{defined}\;as\;1-E/100$ and ΔP is the difference in the pressure caused by the implementation of the mask. [6]

Conventional respiratory masks, say N95, are mainly 4–5 layered systems having nonwoven fibers of polypropylene (PPY) as an active layer [13]. The electro-/melt-spun fibers of PPY are microporous structures and also may hold electrostatic charge gained via the process of development [14, 15]. Thus, along with the size-assisted filtration by the pore and inertial impaction, the electrostatic attraction-assisted filtration is also found to be occurring in PPY layers. Such masks have efficiency at the 95% level as respiratory masks to protect even from virus and bacteria [16]. But, it has been found that the charge on the PPY fabrics does not hold for more than 8 h upon exposure to the ambient conditions and hence, such masks drastically decrease in efficiency after such a short period [17]. Though by design, N95 masks are made for single use, in the present scenario of high demand and scarcity, there are efforts to reuse them [18]. With several methods being tried, a team of scientists has put together the techniques for decontamination and reuse of N95 masks based on experimental data and following the guidelines issued by the Center for Disease Control (CDC), USA [19]. There have also been attempts to recharge the N95 masks post decontamination for better filtration efficiency [8]. Masks made of rechargeable, reusable material have gained attention in the recent past and a few types of such chargeable mask are reported by different groups [3, 8, 20]. Further, a simple mechanical energy-assisted charge restoration post decontamination will be an ideal user-friendly solution for the next generation of economically viable masks affordable to all types of the socio-economic classes of humanity.

Electrostatic charges can be induced in many dielectric systems and certain metals by various methods including plasma discharge [21], ionization by a beam of charged particles [22] and triboelectric charge injection is a useful method for this. The amount of mechanically induced static charge among two systems will depend on their position in the triboelectric series [23] and further apart in position in this series, the pair of system works better as a triboelectric generator (higher charge and hence potential difference) [23]. Fluorinated polymers represent the highest electronegative systems in this series, and polymers such as polyvinylidene fluoride (PVDF) and polytetrafluoroethylene (Teflon, PTFE) belong to this category. These materials have higher triboelectric negative charge generation capacity than PPY—based on their position in the triboelectric series [23]. Wang *et al.* have shown that self-powered electrostatic attraction-based separation-assisted face masks can be developed using PVDF-based nonwoven fabrics [24]. Popular textile materials such as nylon and silk represent positive triboelectric materials. Hence, the combination of such systems may constitute a novel triboelectric generator (TEG) and eventually will be useful in developing facial respiratory masks.

Incorporation of nanoparticle in the friction layers has been proposed to enhance the output of TEG [25] and graphene oxide (GO) has been identified as a negative charge making layer having oxygen functionalities in honeycomb graphene lattice [26]. Further, the oxygen functionalities in GO can ensure adhesion to the surface it is incorporated in and it is important in developing stable coating formulations, such as purification membranes or filters. Such a GO coating was found to be effective even in water purification, as discovered by one of the authors [27] where they have shown that GO coated silica sands can be used as efficient water filter systems that work even against metal ion impurities. Further, the high concentration [30 weight (wt)%] peroxide (hydrogen peroxide, H_2_O_2_) assisted chemical development of GO makes it possible to disinfect the membranes using <10 wt% H_2_O_2_ assisted disinfection, a popular method in practice toward the disinfections of membranes and other surfaces. The commercial availability of GO also makes it useful as an additive in the TEG for triboelectric nanogenerator (TENG) [25]. Recent studies show that GO [28] and other graphene derivatives have high anti-bacterial activity [29], along with other functional properties [29], where those make them as ideal systems for respirator mask applications.

Direct use of PVDF or PTFE as a casted film will affect the air permeation in the membrane and hence the quality factor and is also expensive. It is suggested to use this as a modifier to the PPY nonwoven fabric backbone. Moreover, self-assembled GO membranes are proposed as efficient filtration membranes in different fields [30, 31]. Combining these ideas, we propose here a novel low-cost TENG-assisted mask design with conventional mask materials such as PPY nonwoven fabric, modified with optimum amount containing PVDF and GO mixture and nylon. A tri-layer mask is shown here for its efficacy toward small particle filtration (<0.5 µm) with triboelectric rechargeability, while having efficient permeation capacity to the air. The modified PPY fabrics have higher water contact angle (WCA) ensuring the efficient stopping capacity toward water droplets. The third layer will be cotton, a material positioned at the neutral point in the triboelectric series [23], where it will be in contact with the body to ensure the comfortable wearability of the mask. The cost analyses also show the commercial applicability of these proposed rechargeable masks and the details are discussed in the later part of the article.

Further a simple, easy to implement design of air permeability testing equipment is also discussed in this article, which can be used to test the efficacy of any membrane toward air/gas permeation (via monitoring pressure), humidity (RH, %) and particle size using simple sensors available in the market.

The advantages of GO and PVDF modified PPY fabrics in terms of the charge/voltage created by mechanical stress, charge retention timescales, filtration efficiency (E) and quality factor (Q) are demonstrated by careful experimentation in the following sections.

## MATERIALS AND METHODS

### Materials

The following chemicals and materials are being used for the present investigation: GO powder (>99%, thickness: 1–5 nm) purchased from Platonic Nanotech. PVDF powder from Sigma Aldrich, isopropyl alcohol (IPA) (99%) from SRL Pvt. Ltd. India, and molybdenum disulfide (MoS_2_) powder from Sigma Aldrich (98%, <2 µm). Nonwoven melt-blown PPY (20 GSM) is modified with the PVDF and GO containing paste, as explained below.

### Fabrication of GO and PVDF modified PPY

The paste, consisting of PVDF and GO, used for the modification of PPY mono is prepared by dissolving 100 mg of PVDF and GO in 10 ml of IPA. Initially, PVDF is dissolved in IPA by continuous stirring at ∼600 rpm at 40°C for 1 h. Further, 100 mg of GO is added to it and stirring is continued for another 3 h until it forms a uniform paste. The paste is then uniformly coated over a melt-blown PPY (15 cm × 10 cm) sheet using a simple solution casting method or using a doctor-blade applicator, and is dried at room temperature for 12 h. This film called PPDFGO is sandwiched between a layer of cotton and nylon fabrics commercially purchased to form the tri-layer mask.

### Triboelectric charge testing and air permeability testing

**Figure 1:**
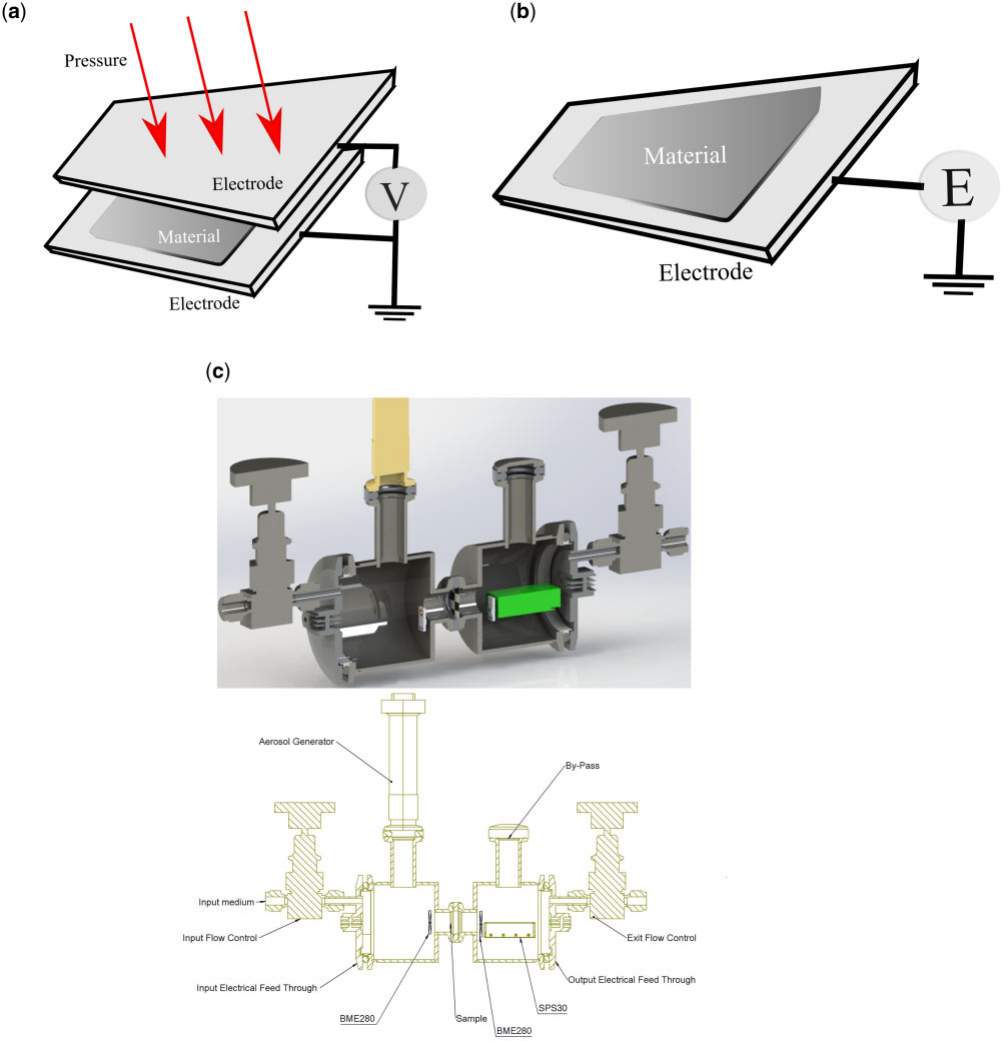
(**a**) Schematic of the triboelectric voltage measurement set up. The ‘material’ is the membrane being tested. Oscilloscope (Model: TEKTRONIX TBS1102B) was used to record the voltage generated via triboelectricity. (**b**) Schematic representation of the charge measurements using an analog electrometer (Model: KEITHLEY 610C). (**c**) Schematics of the membrane testing system: (top) cross-sectional 3D view and (bottom) block diagram showing the different parts. Aerosol generator is a mesh nebulizer. BME 280 is a pressure, humidity and temperature sensor. SPS30 is an optical dust particle sensor. A small air circulating pump is attached at the end flow control value (not shown in picture) that sucks in the ambient air through the test chambers. A gas line can be connected to the inlet value to check for permeability to specific gas flows.

The triboelectric chargeability testing of the membranes is carried out by multiple methods. The triboelectric charge/voltage creation on the materials is also (other than simple finger tapping) measured with controlled force (stress) using a Dynamical Mechanical analyzer (DMA). For this measurement, the membrane is placed between electrodes as shown in Fig. 1a. Voltages produced with minimal impact equivalent to pressing between the palms are recorded using an oscilloscope (Model: TEKTRONIX TBS1102B). In addition, the created charge is monitored over a period of time starting from the time of initial pressing to determine the charge retention capability of the material using an electrometer (Model: KEITHLEY 610C). The electrical measurement set up shown in Fig. 1b is used to detect the charge on the membrane for up to 5 days while keeping the membrane in ambient conditions of temperature and humidity.

The moisture and particle permeability of the membranes are characterized using our homemade set up described in the results section. The system measures the pressure and humidity difference across the membranes in the presence of airflow of about 30–35 L/min as measured by a gas flow sensor. Aerosols containing MoS_2_ particles (1 mg/mL) of size <10 μm are introduced at the inlet side and the number density distribution for various particle sizes measured at the outlet side.

## RESULTS AND DISCUSSION

A simple and rugged in-house device is developed to test the permeability and filtration efficiencies of the membrane. The device contains sensors for measuring pressure, humidity and temperature difference across the membrane barrier, along with the distribution of particle/aerosol sizes passing through the test chamber. The system consists of two chambers (named, inlet and outlet chambers) separated by a port for inserting the membrane under test (Fig. 1c). Ambient air is leaked into the inlet chamber by a valve and pumped out at the exit valve in the outlet chamber. The membrane, with O-ring seals, functionally separates the inlet which is exposed to ambient conditions from the outlet, simulating conditions representing the environment and the respiratory system. BME280 sensors (pressure, humidity and temperature sensor from Bosch Sensortec) are placed on either chamber. Aerosols containing particles of nano to micron sizes are introduced using a nebulizer (Make: BPL Medical Technology, Model: N10) at the inlet side. A Sensirion SPS30 particle sensor was placed in the output chamber to measure the particle density of the aerosols that reach the outlet chamber through the separator. We record the number density of particles in size bins of 0.3–0.5 μm, 0.5–1 μm, 1–2.5 μm, 2.5–4 μm and 4–10 μm and note the change as we introduce the aerosols. All the sensors are interfaced with an Arduino microcontroller board that reads the measured values and stores them in a PC. The setup is tested with membranes from standard N95 masks too and the data are shown in Supplementary Fig. S1. The >95% filtration efficacy of the N95 mask for the particles <0.5 μm is evident from the figure, indicating the proper functioning of the testing system.

**Figure 2:**
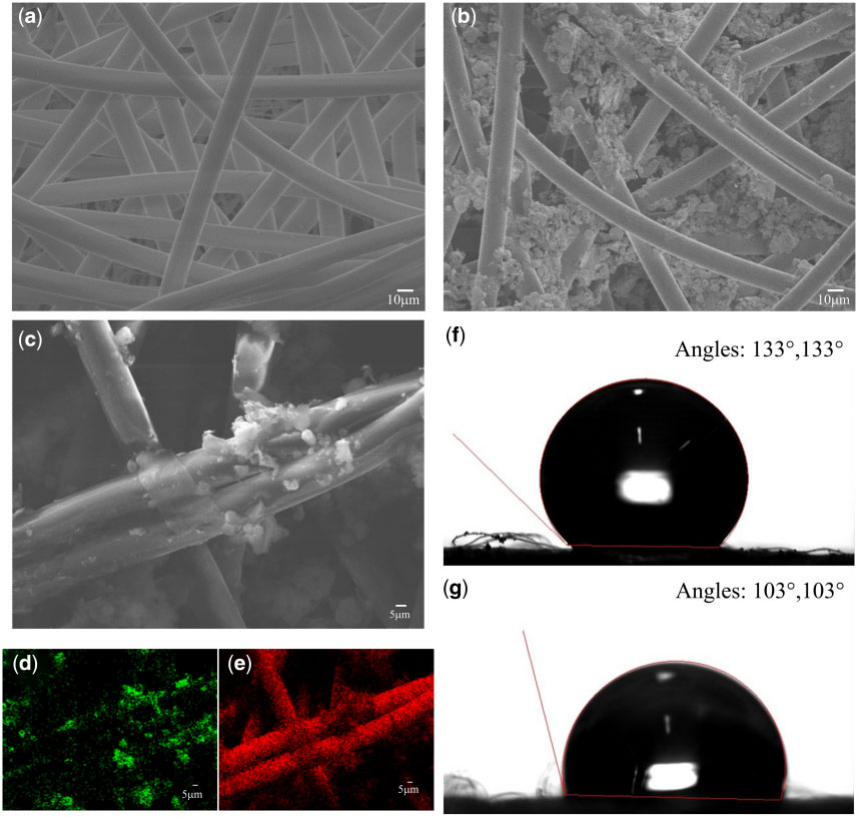
FESEM images of (**a**) melt-spun PPY membrane (PPY monolayer) and (**b**) PPY modified with PVDFGO paste (PPDFGO monolayer). (**c**–**e**) High-resolution SEM image (c) of PPDFGO monolayer and the EDS mapping of the area showing fluorine (d) and carbon distribution (e). The presence of fluorine indicates the presence of PVDF. (**f** and **g**) are room temperature WCA PPDFGO monolayer and PPY monolayer, respectively, indicating the hydrophobicity of the films. Both the left and right contact angles measured are mentioned in the figure.

The melt-spun PPY filter recovered from a commercial N95 mask is used for the present studies. The field emission-scanning electron microscope (FESEM) image of the membrane is shown in Fig. 2a. Nonwoven PPY fibers of 5–10 μm size can be seen in the figure and these membranes are named as PPY mono. The PVDFGO paste modification on these PPY mono films is conducted, as explained in the experimental section, and the FESEM image of such a membrane (PPDFGO mono) is shown in Fig. 2b. The high-resolution SEM image of the PVDFGO paste modified PPY fibers is shown in Fig. 2c and the presence of PVDF is evident from the elemental mapping (EDS) conducted (d and e). The presence of GO is confirmed from the micro-Raman analyses (as shown in Supplementary Fig. S2a). Further, the X-ray diffraction (XRD) data of PPY and the PPDFGO mono are shown in Supplementary Fig. S2b. The diffraction data show the presence of PPY in both the samples. The thin coatings of PVDF and GO in PPDFGO make them below the detection level of XRD (2 wt%). Further, ATR-Fourier Transform Infrared Spectroscopy (ATR-FTIR) measurements are conducted on PPY and PPDFGO (mono) membranes, and the spectra are shown in Supplementary Fig. S2c. The absorption bands at 1167 cm^−1^ and 761 cm^−1^ can be attributed to the symmetrical stretching of -CF_2_ group and in-plane or rocking vibration in α-phase, respectively, indicating the presence of α-PVDF in the PPDFGO [32].

The wettability of the membrane by aerosol is another important measure in its practical long time usage. The PPY mono has a room temperature WCA of 103° while that of PPDFGO mono is found to be 133°, indicating the higher nonwettability from the modified membrane. The higher nonwettability can be attributed to the presence of fluorinated polymers such as PVDF in PPDFGO. When this nonwetting layer is exposed to ambient environment, aerosols will adhere less efficiently to the membrane and will be filtered out from the respiratory system without blocking the membrane pores.

**Figure 3:**
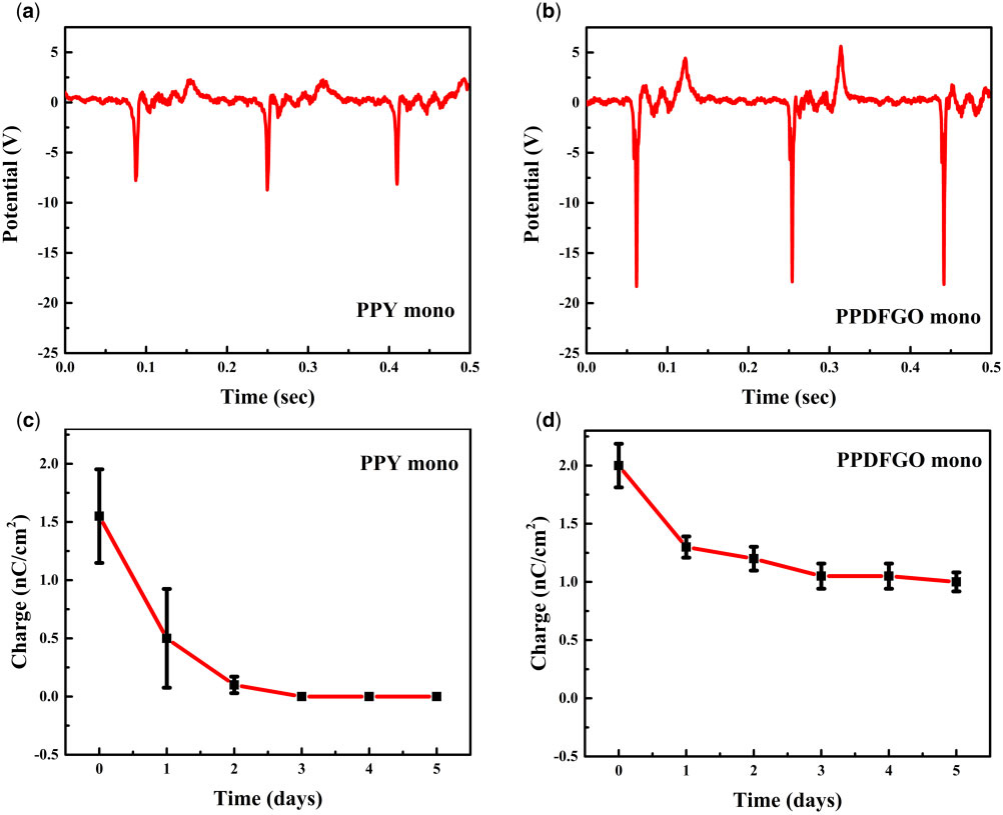
Triboelectric voltage measurement using Oscilloscope (Model: TEKTRONIX TBS1102B) (**a**) and (**b**) indicate triboelectric potential [in volts (V)] generated in PPY mono and PPDFGO mono, respectively. Charge measurements using an analog electrometer (Model: KEITHLEY 610C). The charge measured on PPY monolayer and PPDFGO monolayer after similar mechanical agitation (compression/rubbing with nylon fabric) is shown in (**c**) and (**d**).

The aspects of the membrane that we would like to study to check for its usability in a mask are its charging efficiency, charge retention and its air permeability and particle filtration. Schematics of voltage and charge measurement setups are shown in Fig. 1a and b. The tribocharging of the monolayer PPDFGO is measured using the set ups described in the experimental section, and this is compared with those of PPY monolayer. Figure 3a and b show the voltage generated across the membranes of PPY monolayer and PPDFGO monolayer, respectively. By applying a pressure with the pressing of a thumb, voltages up to 17 V were produced in the PPDGGO monolayer while similar pressure gave rise to about 7 V in PPY monolayer. The charge on the membrane is also measured with an electrometer, and PPY monolayer is found to have an average charge of 1.5 nC/cm^2^ while that on PPDFGO is 2.0 nC/cm^2^. Charge on the PPY is similar to that reported by others [8]. The charge retention while keeping in ambient conditions is monitored over a long period without recharging and the plots of charge as a function of time are shown in Fig. 3c and d for the two types of membrane. It is seen that PPY layer loses its charge in about 2 days, similar to the other reports [18], while the charge on PPDFGO remains for up to 5 days.

**Figure 4:**
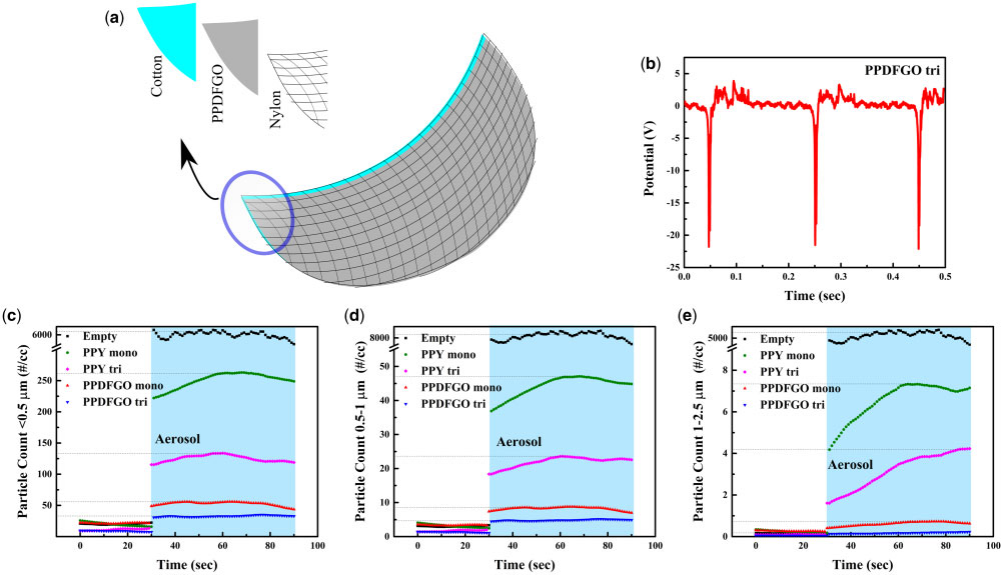
(**a**) Schematic representation of the TENG-based tri-layer mask. (**b**) Triboelectric voltage generated on this tri-layer system measured using a similar set as shown in Fig. 3a. (**c**–**e**) permeation test conducted using particulate materials having sizes <10 μm using different masks (empty represents without any mask in the measurement system discussed before): (c) <0.5 μm (0.3–0.5 μm), (d) 0.5–1 μm and (e) 1–2.5 μm.

A tri-layer TENG mask can be built as displayed in the schematic of Fig. 4a. The tribovoltage generated on such a tri-layer is also studied as in earlier cases and Fig. 4b shows that ≃23 V gets generated across the mask by pressing with thumb.

The particle filtration properties of the membranes, both monolayer and tri-layer, are measured using the homemade set-up, as explained above. The particle counts past the membrane are monitored after introducing aerosol particles. Figure 4c–e shows the plot of particle counts in number/cm^3^ reaching the particle sensor in the absence of membrane (black curve marked as empty), with monolayer PPY (in green), PPY tri (pink, PPY tri is a three-layer system having nylon, pristine PPY and cotton), PPDFGO mono (red) and PPDFGO tri (blue, it is three layers consisting of nylon, PVDFGO coated PPY and cotton), for particles in three sizes ranges (<0.5 μm, 0.5–1 μm and 1–2.5 μm). The sensor also measured particles in 2.5–4 μm and 4–10 μm size ranges and as can be expected those particles are filtered out very effectively even by monolayers (Supplementary Fig. S3). In each plot, the first 30 s show the ambient particle counts and the region shaded in blue is after the introduction of the aerosol particles. The sensor takes a few seconds to reach equilibrium counts after the introduction of the particles and the data are shown until the equilibrium is reached.

**Figure 5:**
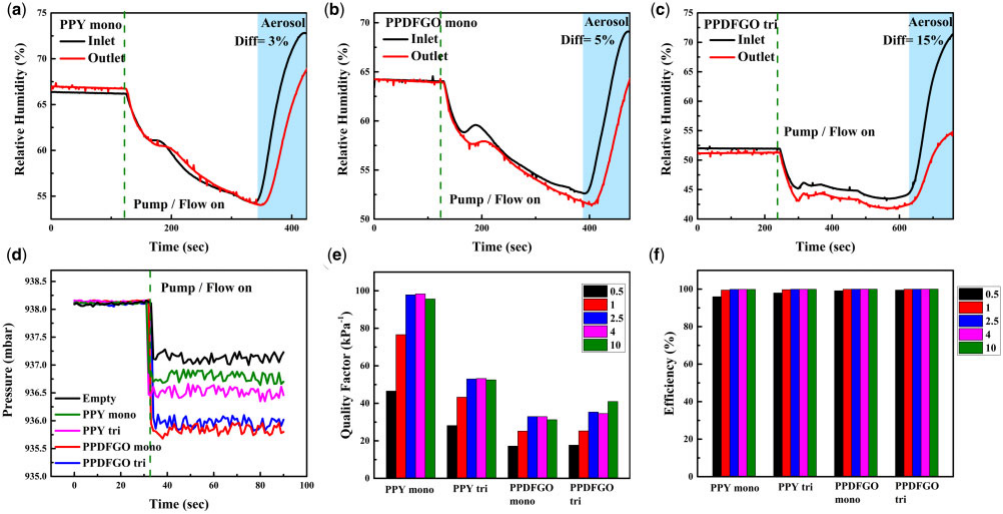
(**a**–**c**) RH in per cent measured at the inlet and outlet side of the system in the presence of monolayer PPY, PPDFGO mono and tri-layer, respectively [the difference (Diff) in the RH% is shown in each figures as 3, 5 and 15%]. Values are plotted before and after switching the pump on and with aerosol introduction at the inlet. (**d**) Pressure at the outlet chamber with and without flow for various membranes separating the inlet and outlet and without any membrane. (**e**) Q estimated based on Figs 4c, d and 5d. (**f**) Filtration efficiency estimated based on Fig. 4c and d.

The horizontal lines in the plots of Fig. 4c–e near each of the membrane type represents the value we take as the equilibrium particle counts when the aerosol generator is on. Based on these values, the filtration efficiency (Fig. 5) is calculated for each of the membranes for different particle sizes, and these data are plotted as histograms in Fig. 5f. All the membranes have a filtration efficiency (E) ≥95%. The PPY material is similar to the ones used in standard N95 masks and has already been shown to give such efficiencies in other studies [4, 6]. The PPDFGO mono has better particle filtration efficiency than PPY as also displayed in Fig. 4c and d where in all three particle size data, the least number of particles permeate the PPDFGO mono and tri-layers.

To further establish the effect of charging in the separation of submicrometer sized particles/aerosols, the injecting aerosol was modified with NaCl (instead of MoS_2_). The filtration efficiencies of the PPDFGO mono after tribocharging (immediately) and 5 days after the charging are calculated, Supplementary Fig. S4. It is evident from the plots that the filtration efficiency for the small-sized (300–500 nm) particles is reduced after 5 days of ambient exposure and it can be related to the decrease in the charge of PPDFGO mono after 5 days of exposure, as shown in Fig. 3d.

The permeability of the membranes is also studied by measuring the pressure and humidity difference across them. The pressure on the downward side (outlet) of the membrane is monitored while the system is being pumped maintaining a flow rate of about 30–35 L/min. Figure 5d shows the pressure measured as function of time with various membranes. The first 30 s show the ambient pressure before the pump is started and as expected, the ambient pressure is the same as the atmospheric pressure when the pump is off. Once the pump is switched on, the pressure drops to a new equilibrium value determined by the flow rate and the membrane permeability. The pressure drop is larger for the tri-layer as compared to monolayer and also the PPDFGO has a larger pressure drop, but the drop is only about 2.5 mbar and would not cause any discomfort or breathlessness if used as a mask.

The relative humidity (RH, %) difference between the down (outlet) and upstream (inlet) sides of the membrane is also monitored with and without flow and in the presence of aerosol particles for monolayer PPY, PPDFGO mono and tri-layer, as shown in Fig. 5a–c. Here the initial times show readings of the sensors in the inlet and outlet side without any pumping/flow. This takes care of any differences in the ambient conditions of the two sensors. Once the flow is switched on, the RH% drops on either side of the system as moisture is removed by the pump. The blue shaded region in the plots (Fig. 5a–c) shows the response of the sensors to the introduction of the aerosol from the nebulizer which is basically water droplets containing particles (MoS_2_ particulate matter of size <10 μm) of differing sizes. It is to be noted that the initial humidity of the chamber may vary with the extent of pumping. The RH% difference between the inlet and outlet increases as the membrane does not allow all the droplets to reach the outlet side of the test system. The RH% difference measured across each of the membranes studied is marked in the corresponding plot. Tri-layer shows the maximum difference in RH of 15%, thus the membrane not only filters particles, but also does not allow moisture to pass through. This is in tune with WCA measurements, as explained before, that PPDFGO has higher hydrophobicity than the PPY.

Based on the filtration efficiency calculated above and the pressure difference measured in Fig. 5d, the quality factor (Q) is estimated for the membranes and is plotted in the histograms, as shown in Fig. 5e. The monolayer PPY shows the largest Q close to 100, as was also shown in other studies [4, 6]. The Q for the tri-layers is lower, but these are all still in the range of 20–40 kPa^−1^ and fall well above the range of values of the common mask materials studied [4, 6]. The PPDFGO tri has a Q value of ∼20 kPa^−1^, indicating its suitability as a respiratory mask [33]. To further establish the role of the nanocomposite paint of GO and PVDF in PPY, PPY membranes modified with PVDF alone and GO alone are also prepared and named as PPDF mono and PPGO mono, respectively. It is found that though the filtration efficiencies of the PPDF mono and PPGO mono (complete coverage) are comparable to that of PPDFGO mono, their Q values are rather poor as compared to that of PPDFGO, Supplementary Fig. S5.

**Figure 6:**
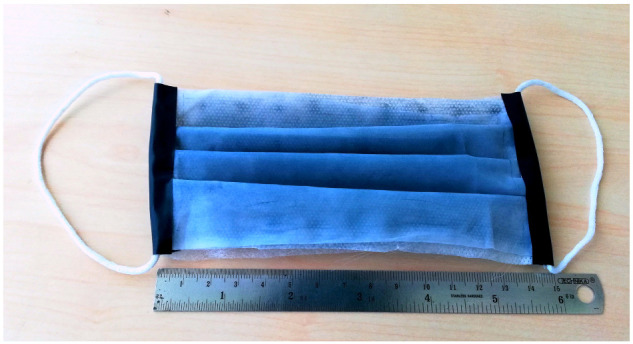
Photograph of the rechargeable PPDFGO tri mask where the three layers are simply stitched together.

Thus, the above results show that PPDFGO tri has the filtration efficiency of >95% and has the Q value of ∼20 kPa^−1^. The PPDFGO tri can be charged using hand bending (or any other small mechanical agitation) as it allows the triboelectric charging among nylon and PVDFGO coated PPY (Fig. 4). In addition, the cost analyses indicate that the price required for the manufacturing of PPDFGO tri is ∼$1 per piece (details of cost analyses are given in Supplementary material). Photograph of a typical PPDFGO tri mask is shown in Fig. 6, indicating the structure and dimensions of a typical commercial mask.

There are different disinfection/sterilization techniques available for the mask and peroxide assisted sterilization is one of the common techniques available [34]. The materials used are chemically stable against H_2_O_2_ (<10% strength) and hence this mask also ensures long-term usability. The coatings, on PPY, made out of GO and hydrophobic PVDF are also found to be chemically as well as thermally (−30°C–70°C, standard mask testing temperature range) stable.

## CONCLUSIONS

A low cost ($1/piece), triboelectric rechargeable respiratory mask is designed and shown for its efficacy toward high filtration efficiency (>95%) and high Q factor (∼20 kPa^−1^). The PPY layer in a conventional N95 mask is modified with PVDF and GO containing ink using a simple solution casting method and such a modification is found to improve the triboelectric voltage and charge (both amount and retention capacity) of the pristine PPY layer. A charge of ∼2 nC/cm^2^ is being generated on PPDFGO and the corresponding voltage is measured as ∼20 V. A simple mechanical agitation, such as hand-bending or sliding one over the other (nylon and PPDFGO mono), is found to be helping to recharge the tri-layer mask system (PPDFGO tri) and this surpasses other external recharging methods of N95 [8], otherwise such masks are inefficient after a small period of exposure (∼8 h). In conclusion, this simple modification of the existing mask design can deliver a long-lasting high filtration efficacy mask, which will be highly useful as a next-generation smart personal protective device affordable to the people in all socioeconomic classes.

## Supplementary Material

itab003_Supplementary_Data

## ACRONYMS

PPY - Polypropylene

PVDF - Polyvinylidene Fluoride

TEG - Tribo Electric Generator

GO - Graphene Oxide

TENG - Tribo -Electric Nano Generator

PPDEFGO- PPY modified with PVDF and GO paste

FESEM - Field Emmision Scanning Electron Microscope

EDS -Energy-dispersive X-ray spectroscopy
