# Supplementary material for: Graphene oxide-based rechargeable respiratory masks
Source: Oxf Open Mater Sci. 2021 Mar 2;1(1):itab003. doi: 10.1093/oxfmat/itab003 (PMC8108635; doi:10.1093/oxfmat/itab003)
Supplement: itab003_Supplementary_Data [file itab003_Supplementary_Data.docx]

**Supporting Information**

**Graphene Oxide based Rechargeable Respiratory Masks**

Stelbin Peter Figerez, Sudeshna Patra, G. Rajalakshmi,* and Tharangattu N. Narayanan*

Tata Institute of Fundamental Research - Hyderabad, Sy. No. 36/P Serilingampally Mandal, Gopanapally Village, Hyderabad - 500046, India.

(* Corresponding authors: raji@tifrh.res.in (G.R.) and tnn@tifrh.res.in (T. N. N.))


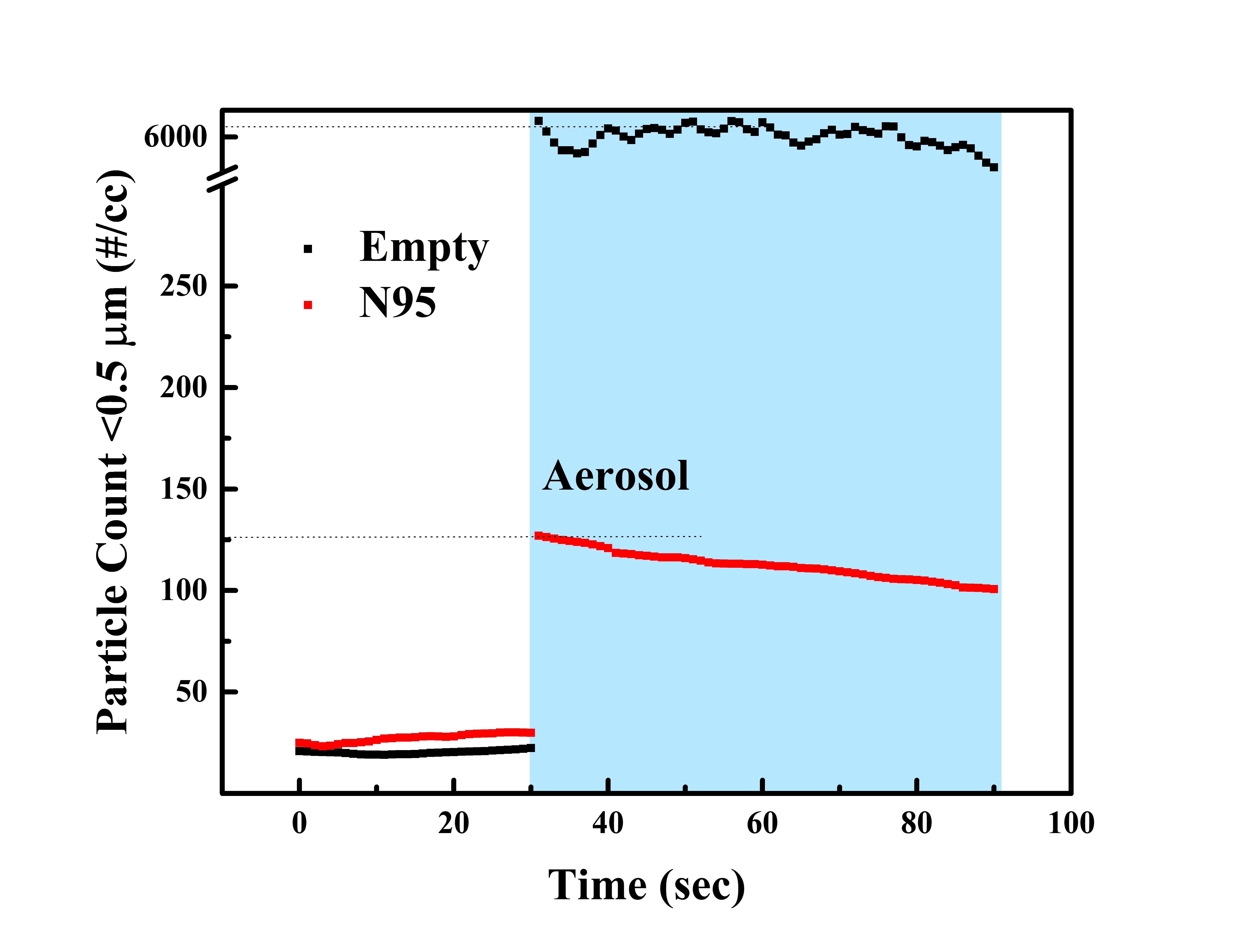


**Fig. S1.** Permeation test conducted using particulate materials having sizes <10 μm for N95 mask using the aforementioned permeability measurement set up: data plotted for < 0.5 μm particle sizes, indicating its efficiency of more than 95%.


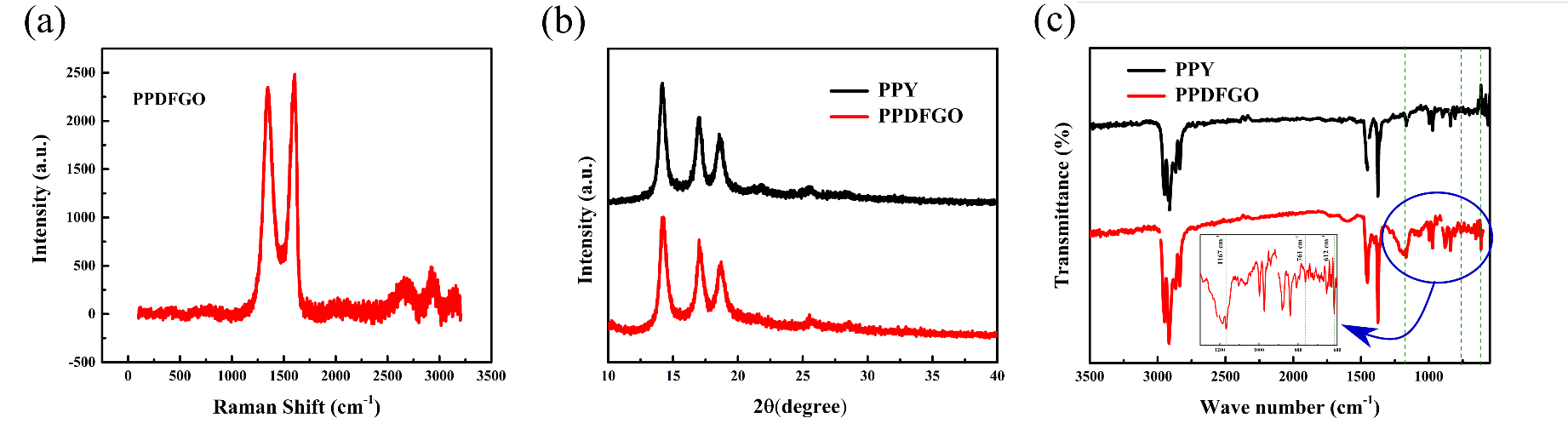


**Fig. S2.**(a)The micro-Raman spectrum of PPDFGO (mono) indicating the presence of GO in the sample. The D (1350 cm^-1^) and G (1580 cm^-1^) vibration modes of graphite are evident from the spectrum. (b, c) XRD and FTIR of PPY and PPDFGO (mono) confirms the α- phase of PVDF.


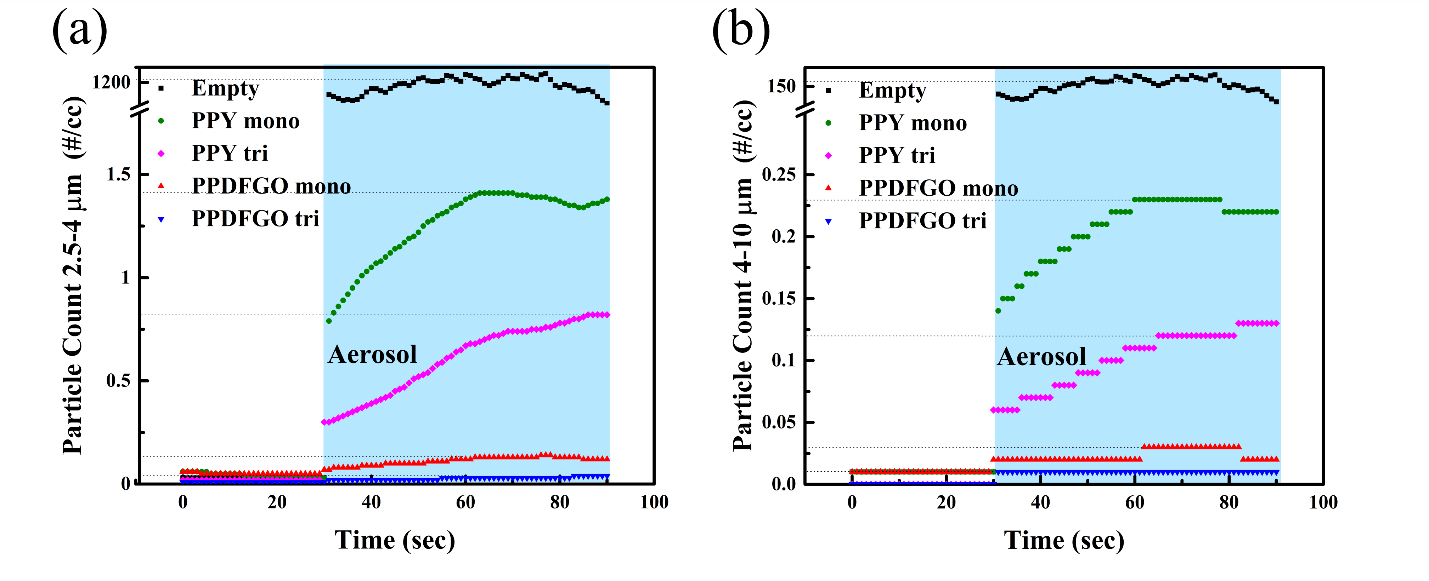


**Fig. S3.** Permeation test conducted using particulate materials having sizes <10 μm using different masks: a) 2.5-4 μm and b) 4-10 μm.

**
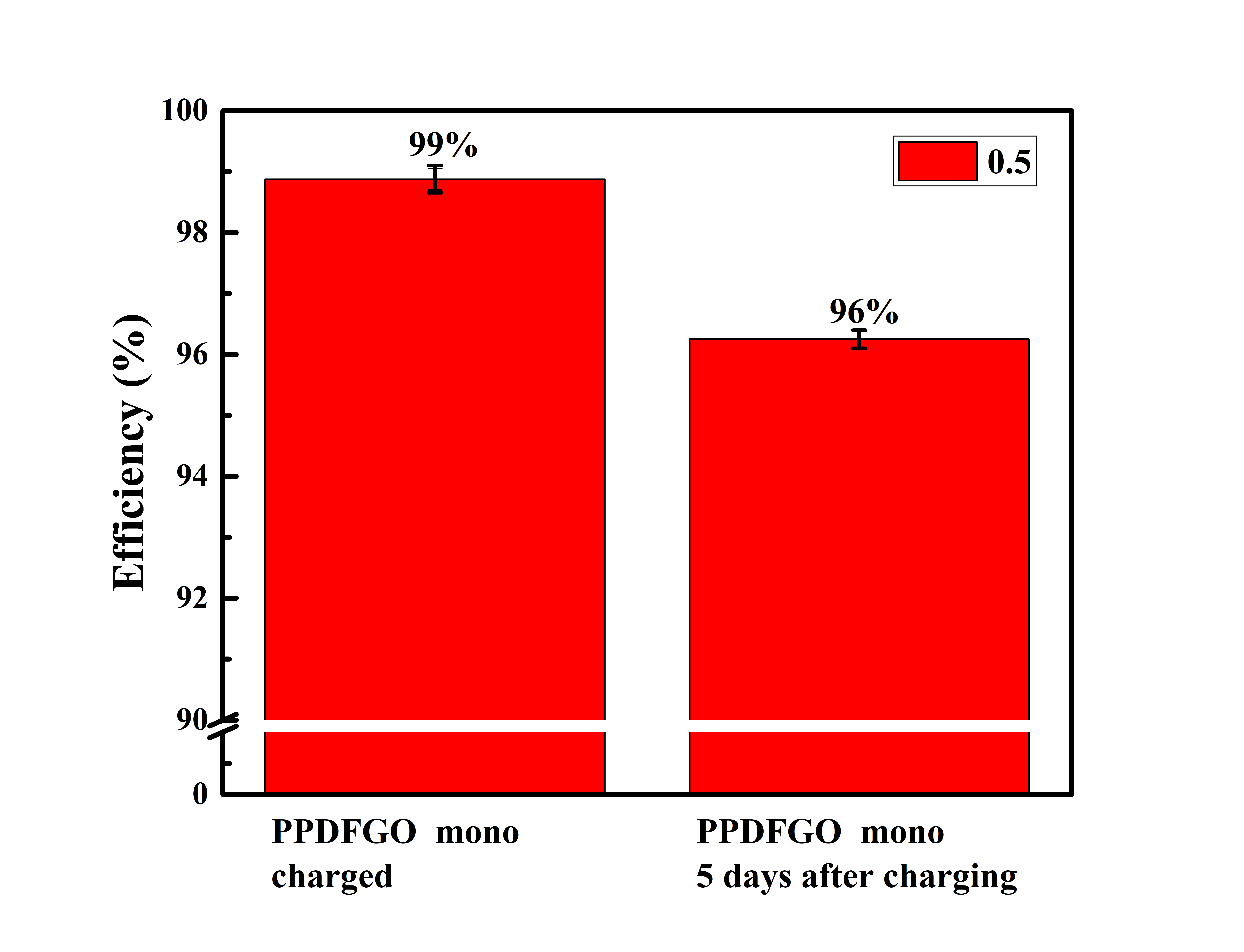
**

**Fig. S4.** The bar diagram indicates the filtration efficiency (for sizes of range 0.3 µm -0.5 µm) of PPDFGO mono just after tribo-charging and after 5 days of charging.

**
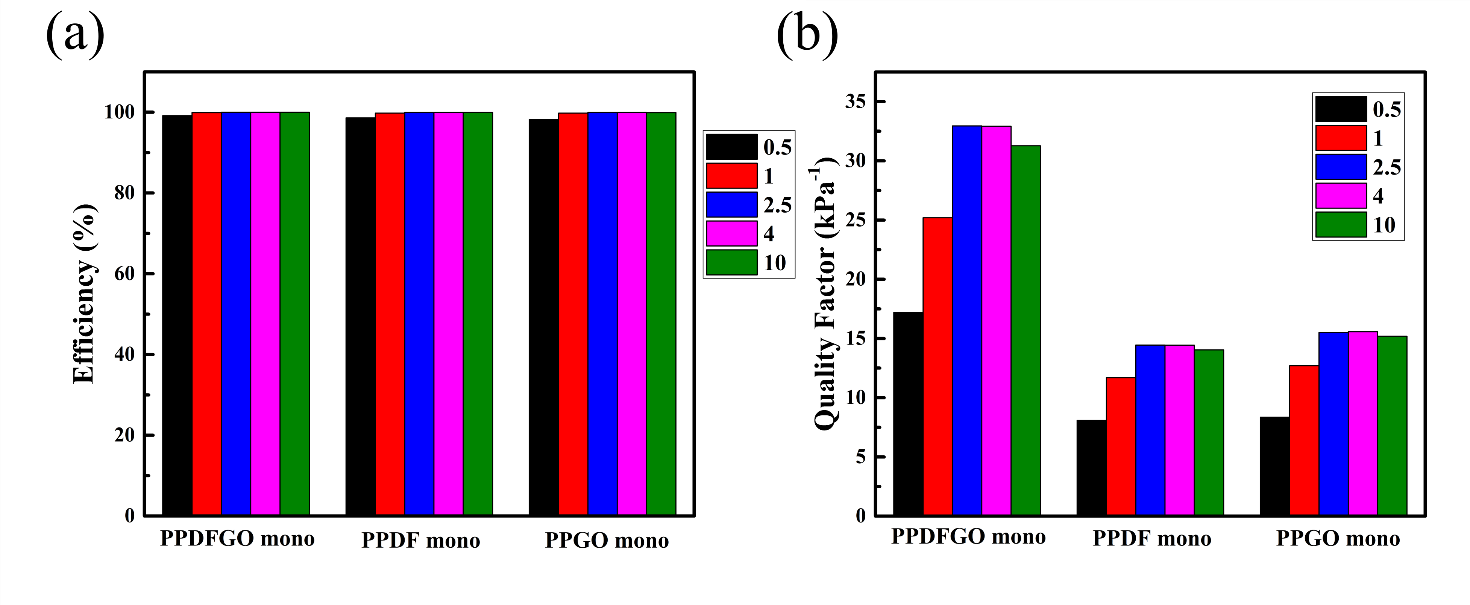
F****ig. S5.** (a) Efficiency and (b) Quality factor of PPDF and PPGO compared with PPDFGO.

**Cost Analyses**:

Ink cost per mask area (15 X 10 cm^2^): 56/- (INR)

[Prices of chemicals are taken from their commercial rate]

Membranes (for tri-layer) Cost: 15/- (INR)

[Commercially available mask was altered for the present study]

Cost of per mask (15 X 10 cm^2^, per piece) ~75/- (INR), including the manufacturing cost.

*Hence, ~$1/piece (mask)*
